# Paracentesis Thoracis

**Published:** 1878-11

**Authors:** S. Logan

**Affiliations:** Professor of Anatomy and Clinical Surgery in the University of Louisiana, &c.


### Miscellaneous.

### Paracentesis Thoracis.

By S. LOGAN, M. D., Professor of Anatomy and Clinical Surgery in the University of Louisiana, &c. (Read before the New Orleans Medical and Surgical Association, July 6th, 1878.)

Since Dieulafoy awakened renewed attention to the method of pneumatic aspiration for the removal of fluid accumulations in the tissues and cavities of the body—a plan really first practiced and published by Bowditch, of Boston—and since Trousseau has been so generally read, the question of the proper treatment of hydrothorax, pneumo-hydro-thorax and pro-thorax (or empyema), has elicited considerable discussion. Nor can we say that definite and generally acknowledged conclusions have yet been reached by the profession.

If we were to listen to the dicta of some of our statistically inclined brethren, whose columns of figures run up into the thousands, we would be obliged to discard all operative procedures and rely exclusively on the Materia Medica of the Therapeutisian and the frequently very clumsy surgery of the Vis Chirurgica Naturæ. On the other hand, did we allow ourselves to be guided by statistical deductions of others—notably M. Dieulafoy—we would hardly approach the bedside of a case of pleurisy without an aspirator under our arm.

The most extensive and at the same time the most elaborately arranged statistical tables, which I have examined, are those published in the British Medical Journal of November 24th, 1877, in an article from the pen of Dr. Wilson Fox, of University College, London. He first gives us one table, "Showing the Mortality per cent. of all cases of pleurisy in different Hospitals" on the continent as well as in Great Britain. This table includes some 13,000 cases. The mean total mortality he estimates as from 10 to 17 per cent. It must be borne in mind that all these were hospital cases, presumably, therefore, coming from less favorable surroundings. The apparently rather high mortality is probably due to this fact.

Dr. Wilson Fox's second table, comprising 2630 cases, is intended to show the "Relative Mortality of Complicated and Uncomplicated Pleurisy;" the mortality in uncomplicated cases ranging from 3 to 7 per cent., while, if I understand the rather obscure paper, the percentage of deaths in the complicated cases ran up to 36.

In the third table is represented, according to the same compiler, the "results of paracentesis in pleurisy" and the mortality percentage is affirmed to "reach as a mean of operation, to 27 per cent. and only falling to or below 10 per cent. in the hands of observers, the large proportion of whose observations have been confined to serious effusions in the early stage, while in others it has ranged as high as 25 to 45 per cent." But he very appropriately adds: "This high mortality, taken both collectively and also in the experience of individual observers, is undoubtedly due in many instances to the nature and to the severity of the cases and of their complications."

Herein truly resides the difficulty in attempting to reach a satisfactory solution of this subject through the statistical method. The cases of thoracic accumulations present such a vast difference in the nature of the cause producing them, in the character of the fluid, in the circumstances of the subject, before, during and after treatment; in the previous or supervening complications, etc., etc., as to render it impossible to draw safe conclusions even with the most careful analysis of the cases. I will not, therefore, trouble you with further statistical details and have only given this summary of the statistics collected by Dr. Fox as a fair sample of many others. Of course we must expect that the "cases operated on" will always present a higher percentage of mortality. The very fact that they have seemed to demand operative procedure is the very best evidence of their graver character.

After the most careful study of all the statistics which can be found the physician or surgeon, when brought face to face with a serious case of thoracic effusion, must in the end decide for himself in each case on its individual merits, the important question as to whether he should resort to surgical measures of relief.

To inform his judgment for such an emergency he must of course bear in mind the various pathological conditions present as well as their immediate or remote possible or probable tendencies and complications.

Thoracic accumulations are either *purulent* or *non-purulent.* It will be more convenient to consider the latter class first.

### 1st.—Non-Purulent Thoracic Accumulations.

Of these again we have several sub-varieties not only in regard to the precise nature of the fluid, but in regard to the pathological processes on which the accumulation depends.

The following classification will answer our present purpose:

(*a*) Acute serous or sero-fibrinous hydro-thorax.

(*b*) Chronic serous hydro-thorax.

(*c*) Sero-sanguineous hydro thorax.

(*d*) Pneumo-hydro-thorax.

In regard to the applicability of thoracentesis in any of its methods to the first of these sub-divisions; or *acute serous* and sero-fibrinous hydro-thorax, I will have but little to say. All agree that the operation is but seldom needed in such cases. The treatment for the active pleurisy is all that is usually required. Absorption generally takes up the fluid as the inflammation subsides; or if this process be tardy, counter irritation, iodine applications, and in some cases iodide of potassium and mercury, separately or combined, will usually suffice.

Should the accumulation increase so rapidly, however, as by its pressure to endanger the life of the patient by suffocation, then I need hardly say that the fluid should be removed. The best method of doing so will be considered further on.

### 2d.—Chronic Serous Hydro-Thorax.

There are several varieties of this form of hydro-thorax, mostly due to the cause producing the accumulation.

In the first place it may result from a chronic or passive form of pleuritis—what Dr. T. Clifford Allbutt designates as "quick effusive pleurisy." The symptoms of the inflammatory precedents or concomitants may readily escape detection, the fever being slight and the symptoms not at all distressing. The patient perhaps only complains of feeling badly in the evening, and the thermometer shows a degree or two of increased temperature as night comes on. Sometimes the accumulation is so gradual that dyspnœa is not complained of till a quantity of fluid sufficient even to endanger life has already been effused.

It is in the application of the operation to such cases that there seems to be the greatest discord among the authorities. On the one hand we find those who affirm that the resort to tapping should be confined to those cases in which the patient is on the verge of suffocation as a dernier resort to ward off the impending death; while on the other hand a larger, and I think increasing number, advise the removal of the fluid as a therapeutic means to *anticipate* in due time this very danger.

Those who refrain from operating till driven to resort to the trocar by the urgency of the symptoms are apt to dwell on the alleged danger incident as they contend to the operation; while their opponents insist on the danger of a sudden and fatal increase of the fluid in the absence of the attendants and on the injurious effects incident to the continued presence of the fluid in large quantities.

Let us then examine the ground on which each party stands.

In the first place what are the dangers asserted to be incident to the removal of a large quantity of fluid from the pleural cavity, and to what degree may such danger, if admitted, be avoided?

Dr. W. Fox again presents us with an elaborate table "Showing the Causes of Mortality in Paracentesis of Serous Effusions in Pleurisy," which will aid us in this discussion. His table comprises 353 cases of operation for serous effusions with a mortality of 87, or 24 per cent., and the causes of death are given as follows: purulent transformation, 32; tubercles of lungs or elsewhere, 20; acute disease of lung or opposite pleura, 5; malignant disease, 3; grave general complications and miscellaneous, 11.

It seems to me that the heading of this table presents as good an example as can be found of begging the question. Can we really consider for example the deaths under the head of malignant diseases as attributable to the paracentesis? The same question might be asked in regard to those under the head of "tuberculous" and those enumerated under "grave complications of miscellaneous."

It may, for the sake of argument, be admitted that some of the patients with these serious accompaniments of the hydro-thorax may have had their term of life more or less shortened by the removal of the fluid, but surely it is simply a petitio principii to assert—either directly or by the phraseology of the heading of the table—that the operation was the "cause of the mortality" in all or even a majority of the cases.

The same criticism, only in perhaps a less positive form, may be applied to the deaths attributed to "purulent transformation" and to "acute disease of lung or opposite pleura."

What proof have we that these conditions were really brought about as a result of the operation? Is it not more reasonable to assume that the very fact that the cases seemed to demand the resort to operative proceedings, is good evidence that they were of so grave a character as to tend of themselves to the development of serious complications of various kinds.

In regard to purulent transformation, also, we are inclined to agree with the eminent French teacher, Prof. Trousseau, and others, who assert that there is little evidence to connect said transformation with the operation carefully performed, unless indeed in a very few instances. Perhaps a more extended investigation of the subject of the transformation of the serous into a purulent fluid may confirm the assertion recently made by M. Dieulafoy. He states that in all cases of serous hydro-thorax the fluid will contain some red blood corpuscles. If even perfectly devoid of color he finds from 1500 to 2000 red corpuscles to the cubic millemeter of fluid. If there be from 6000 to 7000 in the same quantity, he asserts that there is a great liability to purulent transformation, or even if that number exceeds 2500. But, besides these objections enumerated above by Dr. Fox, there are certain other asserted dangers which are worthy of a few moments discussion. Acute congestion of the lungs with albuminous expectoration, dyspnœa, etc., coming on immediately after the operation is one of these. This is true, does sometimes occur; as, indeed, is admitted by all surgeons of experience. But in the first place may we not in many cases avoid this accident by a more gradual removal of the fluid, taking only a little at a time and watching the result; and in the second place may it not be true as affirmed by many that such cases as are specially liable to this danger are those complicated with serious troubles such as tuberculosis, etc.? Dieulafoy asserts that on investigating the circumstances of all the reported "examples of this class of accidents, I find that they have always been associated with either the immediate evacuation of a large quantity of liquid, or with pleurisies complicated with affections of the heart, bronchitis, tuberculosis, etc.; and the precept is to limit the quantity of liquid extracted at a time, and to proportion this to the complications which may exist."

In regard to this point I can give no personal evidence, as I have never seen this condition come on subsequent to the removal of thoracic fluid. Again, cases have been reported of sudden dangerous and even fatal syncope having been produced by the operation. These cases must certainly be very exceptional—provided the fluid be gradually removed. I have never had the accident to occur in either my own hands or in my presence, and I do not think my experience is exceptionally small as compared with other hospital surgeons.

It must be remembered that sudden death occurs in some cases of large accumulation without any operative procedures. T. Clifford Allbutt reports three such cases as having fallen under his personal observation in one hospital. So it would seem that if this be one of the dangers belonging to paracentesis thoracis, it also appertains to the leaving the hydro-thorax unrelieved. The dislodgement of embola and consequent cerebral trouble, and asphyxia from pulmonary obstruction due to the same cause, have been mentioned as possible consequences of the movement of the heart and large vessels back to their positions as the displacing fluid is withdrawn. Possibly this may be one of the risks incident to the operation, but it is too surely so exceptional an occurrence as not to stand materially in the way of securing any undoubted benefits the operation may offer.

In the next place, then, let us enquire what dangers threaten the patient suffering from large fluid accumulations in the pleural cavities; and this injury will at the same time naturally lead to the consideration of the indications for operating for its removal.

We will leave out of consideration the smaller serous or serosanguineous collections, for no one has yet advocated their immediate removal, and all agree to let them alone, or first endeavor by other agencies to promote their absorption.

If a pleural cavity be full or nearly full of fluid, the lung is compressed; its functional activity is destroyed, and if the condition be allowed to continue its tissues become sodden with the fluid, and its ability to expand lessens with the length of time it remains subjected to this deleterious influence If there still remain any tendency to chronic pleuritis, bands or layers of fibrinous effusions may be thrown out, become more or less organized, and tend to permanently imprison the lung, or parts of it, in its collapsed state. Here then is a serious element of danger in delaying the resort of operative measures.

Again, a patient, one of whose pleural cavities is distended with fluid is in constant danger of sudden death from a possible sudden increase of the fluid when surgical aid may not be at hand. Indeed sudden fatal syncope may occur in the very presence of a physician. I have already mentioned that Allbutt alludes to three such cases occurring in his hospital experience alone, and he also states that several others have occurred in private practice. Prof. E. S. Lewis, of this city informs me of a similar case in his personal experience.

Again, the displacement of the heart in many cases probably leads to such twisting of the large vessels—mostly the veins—that their current is impeded and a tendency to thrombosis is the result. It must be borne in mind that the heart is more or less fixed at its base, being there firmly connected with the lungs and thoracic cellular tissue through the medium of the large vessels. When the heart is displaced by the pleuritic fluid it must, therefore, be tilted in the opposite direction, the apex and body being moved on a pivot represented by the large vessels. Under such circumstances the caliber of these vessels—particularly of the more pliable veins—must be lessened near the heart. May not this be the correct explanation of the œdema of the inferior extremities so often met with in these cases,—as well as of the sudden deaths from embola, producing sudden asphyxia, hemi-plegia and pulmonary gangrene? Indeed we may account also for some of the reported cases of sudden death after the operation in which post-mortem examinations have revealed the results of embola, by bearing in mind that a thrombus, already formed at the obstruction above alluded to, may have become detached on the caliber of the vessel being restored by the heart assuming once more its normal position as the fluid is removed. So that in such cases the long continuance of the cardiac displacement may be more to blame for the results than the operation.

Dr. Henry Barnes in a recent article on this subject suggests another danger. He thinks that the long continued stretching of the pleura may so impair its absorbing power as to account for the necessity in some old cases of often repeated operations. There may be a good deal of truth in this suggestion.

I think I have now stated fairly the position assumed by each party to this controversy. If in so doing I have so expressed myself as to give an intimation of the side to which I belong, it is perhaps because the "common sense of the thing," if I may be permitted the expression, clearly leads to the conclusion that the party who declines to wait till death, so to speak, actually lays his hand on the patient, before taking steps for his relief, is surely right. The patient is in acknowledged risk of his life; the danger is definite and clearly understood. In uncomplicated cases it is due solely to the continued presence in large quantity of the pleuritic effusion; in complicated cases the fluid adds greatly to the danger and may prove the immediate cause of death.

Under such circumstances shall we wait till the patient is cyanosed and gasping for breath, or shall we ward off these extreme symptoms by removing the immediate cause which tends this way.

What then are the indications for operation in cases of chronic serous hydro-thorax.

It is convenient to have some arbitrary rule in this matter, though, as in all medical and surgical cases, each one should be judged by itself, at least within certain limits.

I would therefore say, that as a rule we may be safely guided by the recommendation given by Dr. Barnes, in the article already mentioned. If the pleura be half full he waits to see what time, aided by therapeutic means, may accomplish. If the cavity become two-thirds full, the point of danger approaches and the fluid should be lessened or removed. This rule I would approve as practically a good one.

In cases of acute serous effusions, it is best—unless urgent symptoms present themselves—to wait and see if the remedies used for the inflammatory condition may not suffice. As already stated these effusions are usually absorbed, and the operation is generally unnecessary.

In the other varieties of non-suppurative accumulations, *i. e.*, sero-sanguineous, hydro-thorax and pneumo-hydro-thorax, we must be guided by the exigencies of the case; the indications indeed may be considered the same as in the case of the chronic serous hydro-thorax. I need not therefore delay longer to specially consider the operation as applicable to these forms of pleural trouble.

Nor need we say much on this occasion concerning the application of paracentesis to the relief of the *purulent thoracic accumulations.*

Not but that this is a subject of great importance: there are few more so. But we do not find much difference among modern surgeons in regard to it; and, therefore, after simply stating the conclusions the profession has reached relating thereto, I may leave the subject as "*res adjudicata.*"

A purulent accumulation anywhere in the body is always productive of harm and frequently involves the greatest risk. Whereever practicable it should be removed, and such measures be adopted as will tend to arrest the further production of pus, and to give immediate exit to what may continue to be formed. Empyema affords no exception to this wise surgical axiom. Indeed I need hardly say that there are circumstances connected with this form of suppuration which makes the law specially applicable. The accumulating pus is here specially injurious, and at the same time its removal is perfectly practicable. The almost universal experience of modern surgeons confirms the truth of these assertions, and we may, therefore, without further delay pass on to consider the last phase of our subject.

### THE METHOD OF OPERATING FOR THE REMOVAL OF THORACIC ACCUMULATIONS.

These may be classified as follows, and their special adaptability to the various conditions found will be considered in the order in which they are mentioned.

1st. Simple tapping with trocar and canula; 2d, The syphon method; 3d, Aspiration; 4th, The drainage method.

1st. Simple tapping with a trocar and canula without the antiseptic spray is now but seldom resorted to in cases of serous effusion. So great is the fear of the admission of air influencing the transformation of a serous into a purulent collection, that the aspirator or some other means is fast supplanting this old method. If, however, the antiseptic spray be kept up during the whole time there is no reason why we may not expect very good results in cases where the means of securing a more perfect exclusion of air are not at hand. If practicable, however, I would prefer

2d—THE SYPHON METHOD.

This is really a modification only of the last, and the necessary apparatus is so simple as to be pretty generally capable of being extemporized. An ordinary trocar and canula with a few feet of gutta percha tubing is all that is absolutely required. The canula, however, should have no shoulder, or only a small collar, in order that it may be readily slipped into the tube. If the proximal end of this tube be slipped over the distal end of the canula, the trocar can be made to perforate the side of the tube near the canula and to enter the latter. When this is done, and everything is ready for the operation, sink the whole apparatus under the carbolized solution of about 2 or 3 per cent. Then let an assistant seize the distal end of the tube between the fingers, mashing it so tight as to exclude the air. Let the surgeon now at once puncture the thorax and withdraw the trocar. As the latter is removed the tube may be slipped further over the canula so that the latter may pass beyond the injury inflicted by the trocar where it penetrated the wall of the tube. By causing the assistant now to lower the distal end of the tube and keep it below the surface of the fluid in the basin containing the carbolic solution a perfect syphon is made, and the liquid from the thoracic cavity is conducted under the fluid in the basin. This simple expedient, first suggested by Dr. Girgensohn, of Riga, Prussia, meets every indication for the evacuation of serous fluids. I show you, however, an apparatus which I use in such cases and which presents some conveniences. It consists of a hollow trocar, sharp-pointed and covered with a blunt pointed canula, which, after perforation, may be made to slip forward and cover the sharp point of the trocar. It has the advantage over the ordinary trocar and canula in this: the necessity for the removal of the trocar is obviated and the operation is thereby materially facilitated. It is better than the ordinary aspirator needle in this respect; *i. e.*, the sharp end being protected there is no danger of injury to the parts in the thorax as the fluid subsides and the lung expands. The gutta percha tube is interrupted with glass at one point, thus enabling the operator to see the nature of the fluid as is passes; and there is a metallic spring near the further end by which the tube can be compressed at will, thus obviating the uncertain resort to the fingers of an assistant. The syphon plan has one advantage over aspiration which in some cases is of decided benefit. The power applied in withdrawing the fluid can be regulated more delicately. This is done by raising or lowering the basin in which the distal end of the tube is submerged. In cases of diseased lung, or suspected adhesions, where a very gradual and gentle suction is desirable, I would, therefore, prefer the syphon method.

### 3d—ASPIRATION.

As the most certain method of excluding air while removing the fluid, this plan claims precedence over all others. It is now so familiar to the profession, that I need hardly say any thing more about it. By using the finer tubes the removal of the fluid may be effected as slowly as desirable; and thus the dangers attending rapid and forcible exhaustion of the pleural cavity are in a great measure obviated.

### 4th—DRAINAGE.

The other methods described are only applicable to the non-suppurating effusions. Where pus is present, not only must the cavity be evacuated, but efficient drainage must be secured. This can only be effected by some permanent opening, which at the same time affords us the means of washing out the cavity. There are several methods, each specially adapted to certain cases, which may serve to carry out the indications in view.

In the first place I have in some cases of simple empyema succeeded very well by merely inserting a drainage tube through the large canula by which the pus has been evacuated, using daily injections of carbolic solution for the purpose of assisting in cleaning the cavity of all pus, and at the same time acting antiseptically on the walls.

In other cases, however, a double opening is found advisable. It is usually recommended that we make one of these in the usual position for paracentesis,—*i. e.*, about the 8 or 9 intercostal space on a line with the inferior angle of the scapula,—while the other is made anteriorly.

Chascaignac, and many French surgeons following him, pass a drainage tube through from one opening to the other. I adopted this plan in two cases; but have abandoned it entirely. I now agree with those who assert that the tube thus used acts too much like a seton; and, indeed, it is unnecessary, to say the least. A tube in each opening only sufficiently long, to keep open the orifice through the thoracic walls is all that is required. Nor, as M. Potain has shown—at a recent meeting of the Paris Medical Society on the 27th of last July,—is it at all necessary that the openings should be far apart. He suggests that they be made alongside of each other—so near that should a freer opening become necessary the two may be united by an incision into one, both being located in the sixth intercostal space. In washing out the cavity the fluid used is just as efficiently applied to the whole interior walls as if the openings were far apart. In doing this he uses the syphon method, and as he says the cavity is made to wash itself out.

There is frequently some difficulty in keeping the openings free, on account mainly of the approximation of the ribs in cases where there is a collapse of the thoracic walls. A suggestion, whose authorship has just now escaped me, has recently been made, which

I take to be a good one. It is to use a hollow uterine stem passary in each opening. Its mechanism is such that it retains its position, and the distal ends can be inserted into two gutter-percha tubes, so as to admit of syringing out the cavity or injecting it by the syphon method. But there are some obstinate cases—generally of old standing and neglected—in which a large free opening becomes necessary. Here a free incision should be made between the ribs, and if they are found to have approximated each other so much that the little finger cannot be made to pass between them, a portion of the upper border of the lower-most rib must be removed with a small trephine, or bone nippers. A free opening is thus secured, through which frequent ablutions may be practiced. —*New Orleans Medical Journal.*

